# Distal aortic biomechanics after transcatheter versus surgical aortic valve replacement: a hypothesis generating study

**DOI:** 10.1186/s13019-023-02467-z

**Published:** 2023-11-30

**Authors:** Lisa Q. Rong, William Zheng, Andrew Martinez, Mohammed Rahouma, Richard B. Devereux, Jiwon Kim, Bassam Osman, Maria C. Palumbo, Björn Redfors, Leonard N. Girardi, Jonathan W. Weinsaft, Mario Gaudino

**Affiliations:** 1 Department of Anesthesiology, Weill Cornell Medicine, 525 East 68th Street, New York, NY USA; 2 University of Michigan Medical School, Ann Arbor, USA; 3 Department of Cardiothoracic Surgery, Weill Cornell Medicine, New York, NY USA; 4 Department of Cardiology/Medicine, Weill Cornell Medicine, New York, NY USA; 5 Department of Electronics, Information and Bioengineering, Politecnico di Milano, Milan, Italy; 6 Department of Population Health Sciences, Weill Cornell Medicine, New York, NY USA; 7 Department of Molecular and Clinical Medicine, Institute of Medicine, Gothenburg University, Gothenburg, Sweden; 8 Department of Cardiology, Sahlgrenska University Hospital, Gothenburg, Sweden

**Keywords:** Aortic strain, Transesophageal echocardiography, Surgical aortic valve replacement, Transcatheter aortic valve replacement

## Abstract

**Background:**

Biomechanical effects of transcatheter (TAVR) versus surgical (SAVR) aortic valve interventions on the distal aorta have not been studied. This study utilized global circumferential strain (GCS) to assess post-procedural biomechanics changes in the descending aorta after TAVR versus SAVR.

**Methods:**

Patients undergoing TAVR or SAVR for aortic stenosis were included. Transesophageal (TEE) and transthoracic (TTE) echocardiography short-axis images of the aorta were used to image the descending aorta immediately before and after interventions. Image analysis was performed with two-dimensional speckle tracking echocardiography and dedicated software. Delta GCS was calculated as: post-procedural GCS—pre-procedural GCS. Percentage delta GCS was calculated as: (delta GCS/pre-procedural GCS) × 100.

**Results:**

Eighty patients, 40 TAVR (median age 81 y/o, 40% female) and 40 SAVR (median 72 y/o, 30% female) were included. The post-procedure GCS was significantly higher than the pre-procedural GCS in the TAVR (median 10.7 [interquartile range IQR 4.5, 14.6] vs. 17.0 [IQR 6.1, 20.9], *p *= 0.009) but not in the SAVR group (4.4 [IQR 3.3, 5.3] vs. 4.7 [IQR 3.9, 5.6], *p *= 0.3). The delta GCS and the percentage delta GCS were both significantly higher in the TAVR versus SAVR group (2.8% [IQR 1.4, 6] vs. 0.15% [IQR − 0.6, 1.5], *p *< 0.001; and 28.8% [IQR 14.6%, 64.6%] vs. 4.4% [IQR − 10.6%, 5.6%], *p *= 0.006). Results were consistent after multivariable adjustment for key clinical and hemodynamic characteristics.

**Conclusions:**

After TAVR, there was a significantly larger increase in GCS in the distal aorta compared to SAVR. This may impact descending aortic remodeling and long-term risk of aortic events.

**Supplementary Information:**

The online version contains supplementary material available at 10.1186/s13019-023-02467-z.

## Introduction

Surgical aortic valve replacement (SAVR) and transcatheter aortic valve replacement (TAVR) are the standard treatments for severe aortic stenosis (AS). Previous studies have demonstrated similar efficacy and short- to mid-term outcomes for patients who undergo SAVR and TAVR [1–5] but the differential impact of the two procedures on aortic biomechanics is not well understood. Aortic stenosis can be associated with concurrent aortopathy, and changes in aortic energy propagation after SAVR versus TAVR may affect distal aneurysm growth and aortic disease progression particularly in patients with genetic aortopathies or bicuspid aortic valve (BAV).

Global circumferential aortic strain (GCS) is a measure of arterial stiffness and aortic biomechanics that can derived from standard two-dimensional echocardiographic images of the aorta [6–9]. This measure can assess the deformation of the aortic wall between end-diastole and peak-systole and has been validated against the gold standard cardiac magnetic resonance [7, 10]. Previous studies have used GCS to evaluate distal aortic biomechanics after aortic valve and ascending aorta replacement in cardiac surgical patients. However, changes in distal aortic biomechanics after TAVR and SAVR have yet to be characterized. This hypothesis-generating study utilized echo-derived GCS to assess changes in distal aortic biomechanics after TAVR and SAVR. We hypothesized that TAVR would increase GCS compared to SAVR due to the larger device footprint compared to the bioprosthetic surgical valves.

## Methods

The study was approved by the Weill Cornell Institutional Review Board (IRB number 20-0102134). Patients that underwent TAVR or SAVR for severe isolated aortic stenosis were prospectively enrolled and consented between January 2021 and March 2023. This was a hypothesis generating study, and no formal sample size calculation was performed. All patients met clinical criteria for aortic valve intervention due to severe AS as determined by a multidisciplinary team of cardiac surgeons, cardiologists, and echocardiographers. Patients with more than mild valvular regurgitation (aortic, tricuspid, or mitral) were excluded, as were those aborted intervention (TAVR), and with inadequate imaging for strain analysis were excluded.

SAVR was performed through full sternotomy and cardiopulmonary bypass using bioprosthetic valves, with size and valve type listed in Table 1.

**Table 1:** Population characteristics

	Overall (n = 80)	SAVR (n = 40)	TAVR (n = 40)	*p*
*Clinical characteristics*				
Age [year (median IQR)]	76.5 [70,83]	72 [63,75]	81 [74,88]	< 0.001
Blood pressure (mm Hg)				
Male gender	52 (65)	28 (70)	24 (60)	0.48
Bicuspid aortic valve	16 (20)	13 (32.5)	3 (7.5)	0.012
Pre-AVR AS severity				1
Moderate	1 (1.2)	1 (2.5)	0	
Severe	79 (98.8)	39 (97.5)	40 (100)	
*Clinical risk factors*				
Coronary artery disease	32 (40)	12 (30)	20 (50)	0.11
Hypertension	57 (71.2)	26 (65)	31 (77.5)	0.32
Hyperlipidemia	51 (63.7)	24 (60)	27 (67.5)	0.64
Diabetes mellitus	18 (22.5)	8 (20)	10 (25)	0.77
NYHA ≥ 2	68 (85)	29 (72.5)	39 (97.5)	0.005
*Intraoperative data*				
Valve size (mm, median [IQR])	25 [23,26]	23 [23,25]	26 [23,29]	< 0.001
Edwards (Magna/Inspiris resilia; Sapien 3)		33 (82.5)	35 (87.5)	
Medtronic (Mosaic Valve/Avalus; Core valve)		7 (17.5)	5 (12.5)	
Mean gradient				
Pre-AVR	40.5 [30.2,47]	38.8 [29.8,45.5]	41 [33.6,47.7]	0.33
post-AVR	6 [4, 9]	8 [5.5,11]	4 [3, 6]	< 0.001
Pulse pressure				
Use of inotropes*	29 (36.2)	27 (67.5)	2 (5)	< 0.001
Use of vasopressors**	23 (29.1)	9 (23.1)	14 (35)	0.36

*AVR* Aortic valve replacement, *IQR* Inter-quartile range, *NYHA* New York Heart Failure Association, *SAVR* Surgical aortic valve replacement, *TAVR* Transcatheter aortic valve replacement

*Inotropes = epinephrine, milrinone, dobutamine infusions

**Vasopressors = vasopressin, norepinephrine, phenylephrine infusions

All intraoperative transesophageal echocardiography (TEE) and/or transthoracic echocardiography (TTE) images were prospectively acquired via a standard imaging protocol using clinical equipment (EPIQ 7, Philips Medical Systems [Andover, MA] ultrasound systems). For SAVR, intraoperative TEE images were captured with stable hemodynamics immediately after induction and before incision (pre-procedure), and after chest closure (post-procedure) as previously described [6]. For TAVR, TEE and TTE echo exams were performed with stable hemodynamics before procedure start (pre-procedure), and after valve deployment (post-procedure). Hemodynamic variables including cardiac output and cardiac index were obtained at the time of the echo exams via a pulmonary artery catheter. Pulse pressure was derived from the arterial blood pressure.

### Aortic global circumferential strain

TEE and TTE images were used to capture short-axis images of the descending aorta before and after valve replacement. Images were analyzed by two experts blinded to patient data but not to modality. GCS was used to measure the change in circumferential deformation in the aortic short axis images using speckle-tracking of the aortic wall using dedicated software (Qlab version 10.8.5, Philips Healthcare, Amsterdam, Netherlands) as previously described [6]. Briefly, a center point was placed within the aorta in addition to an inner and outer circle that approximated the endothelial and adventitial surfaces of the aorta during the end-diastolic frame, allowing for tracking of the aortic wall throughout the cardiac cycle, and the width was adjusted to contain the entire wall thickness of the aorta. Automatically generated strain measurements were then evaluated for optimal border tracking. If the software output did not adequately track the aortic wall and/or the generated strain curve was not smooth, the inner and outer circles were adjusted circumferentially in the end-diastolic and end-systolic frames to enhance border tracking [6, 9, 11].

The 6 sub-segments of the aorta were averaged to measure the following aortic biomechanical variables:

- *Global circumferential aortic strain (GCS)* maximal deformation of the aortic circumference between systole and diastole (measured as the relative (%) difference between these two time points; [end-systole–end-diastole]/end-diastole*100).
- *Change in GCS (Delta GCS)* post-procedure GCS-pre-procedure GCS.
- *Relative change in GCS (Percent Change GCS)* post-procedure GCS-pre-procedure GCS/pre-procedure GCS × 100
- *Pulse-Pressure Adjusted GCS* GCS divided by pulse pressure (PP):(GCS/PP)
- *Time to peak (TTP) strain* calculated as time interval between end-diastole (aortic valve opening) and average peak global circumferential aortic strain.

Aortic end-systolic area (ESA) and end-diastolic area (EDA) were also measured and used to calculate fractional area change (FAC) of the aorta (FAC = [ESA − EDA]/ESA). Aortic distensibility index was calculated via well-validated method; [(ESA − EDA)/(ESA)(PP)] [12].

**Fig. 1:**
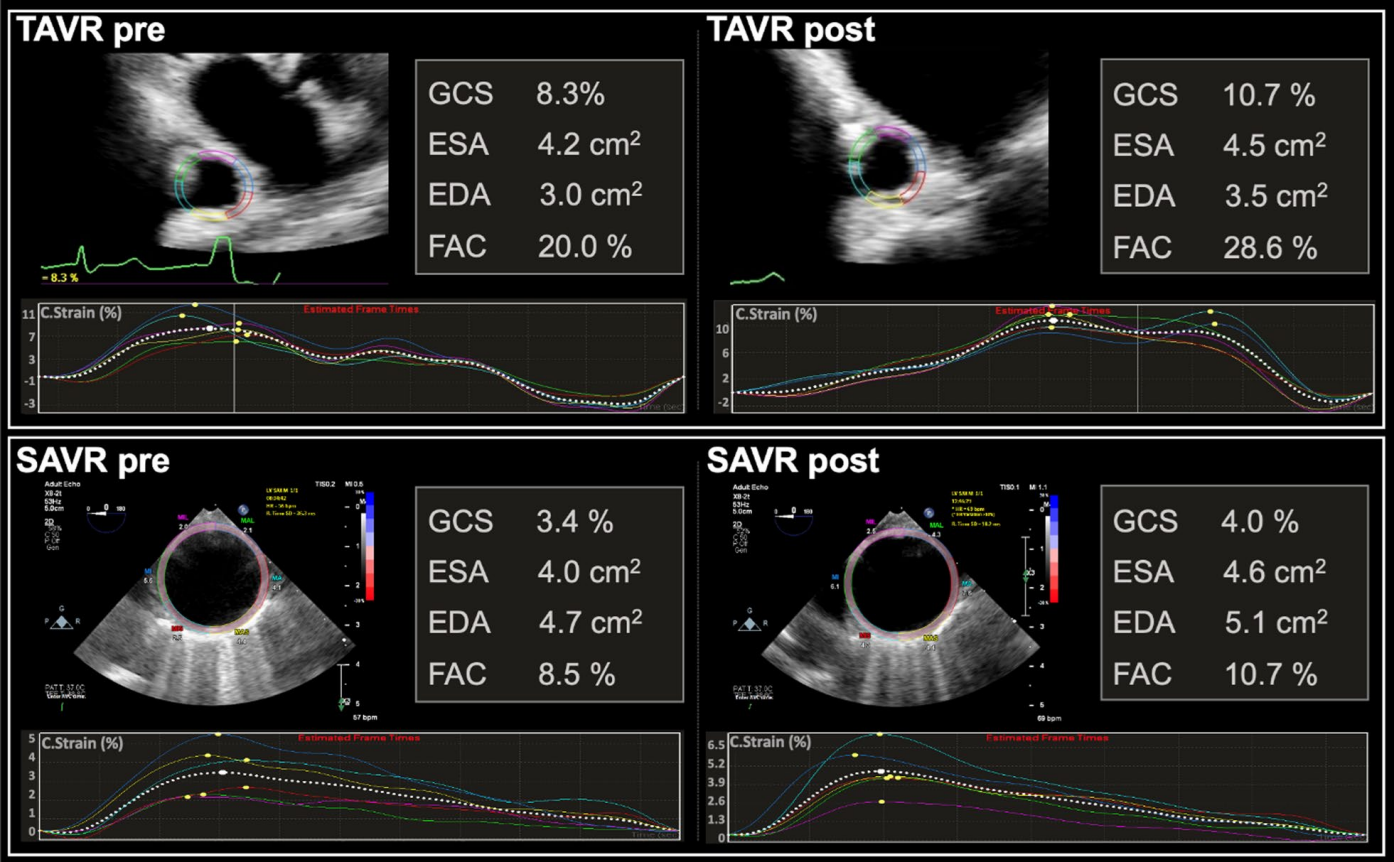
Representative example of a patient who underwent TAVR, and SAVR, and their pre-procedural and post-procedure aortic strain measurements. EDA, end diastolic area; ESA, end systolic area; FAC, fractional area change; GCS, Global circumferential strain; SAVR, surgical aortic valve replacement; TAVR, transcatheter aortic valve replacement

Figure 1 provides a representative example of aortic analyses performed pre- and post-SAVR and TAVR.

### Statistical analyses

Categorical variables were reported as counts and percentages. Normality of continuous variables was determined by the Shapiro-Wilkinson test. Normally distributed data were reported as mean ± standard deviation (SD) and non-normally distributed variables were reported as median and interquartile ranges (IQR).

The Student’s t-test was employed to assess differences between groups with normally distributed variables and the Mann–Whitney U test was used if variables were non-normally distributed. Chi squared and Fisher’s exact tests were used for categorical variables, as appropriate.

To adjust for potential differences in baseline characteristics and imaging methods between patients, analysis of covariance (ANCOVA) was used to assess the difference of delta GCS and percentage change GCS between groups. Adjustment for key baseline and hemodynamic variables included age, sex, baseline GCS, valve type [bicuspid, tricuspid], NYHA class, use of inotropes/vasopressors, preoperative systolic and diastolic blood pressure, preoperative CI and postoperative CI, preoperative aortic stenosis severity, and post-procedure aortic valve mean gradient. Results of ANCOVA were expressed as mean and standard error and visually as effect plots that showed mean and 95% confidence intervals.

Two-sided *p *< 0.05 was considered statistically significant without multiplicity adjustment. Analysis was performed using R version 3.6.1 within RStudio. Two sensitivity analyses were performed: (1) repeating the main analyses including only patients with trileaflet aortic valve (BAV patients were excluded to reduce possible heterogeneity due to valve morphology) and (2) adding imaging modality (TEE vs. TEE) to the ANCOVA multivariable analysis (to reduce possible heterogeneity due to different imaging techniques).

## Results

**Fig. 2:**
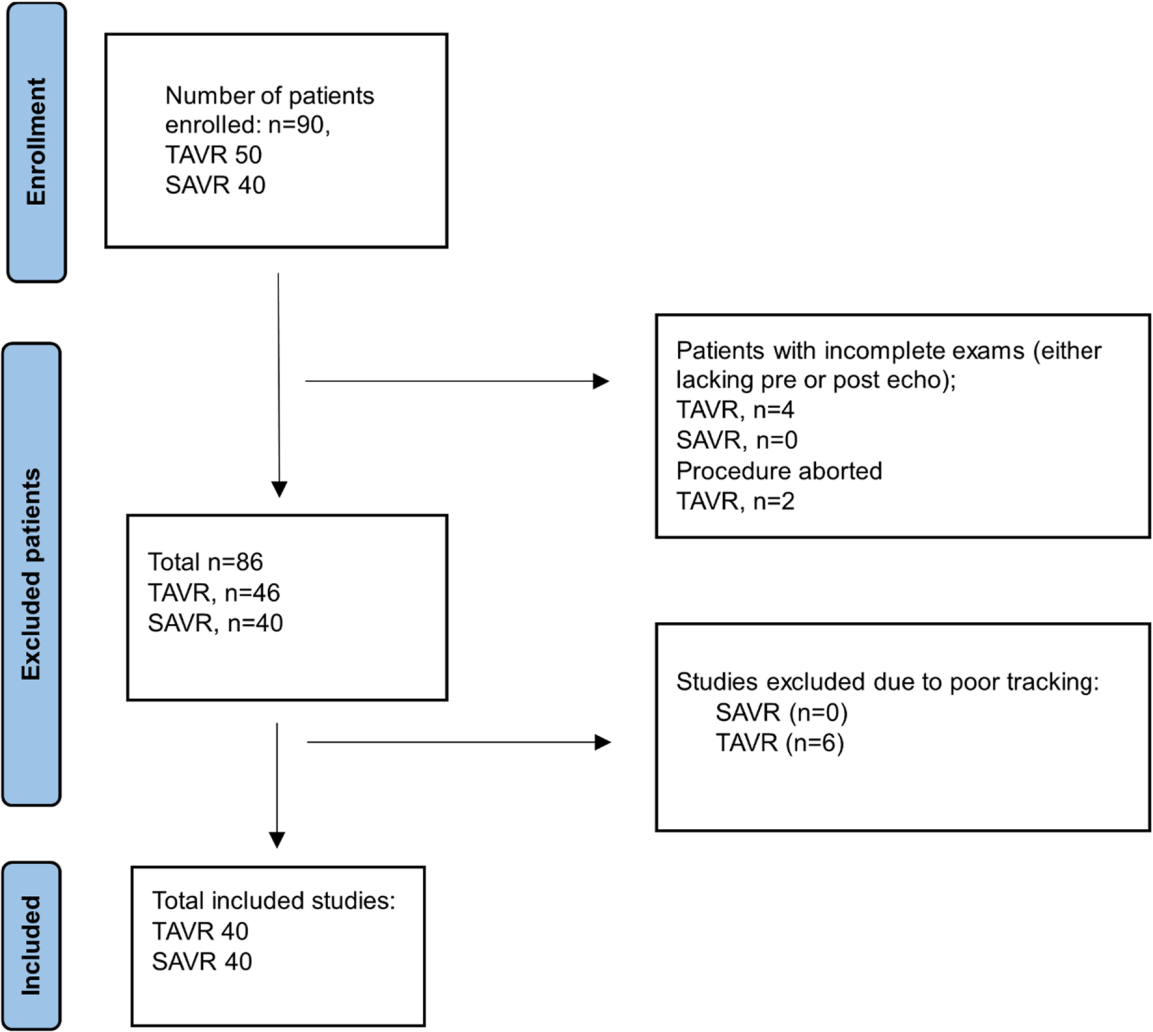
CONSORT diagram

Eighty patients (40 TAVR and 40 SAVR) were included in the study cohort (see Fig. 2 for details of patient inclusion); the median age of the included patients was 76.5 years (IQR 70–83), 65% were men, 20% had bicuspid aortic valves. Baseline and intra-procedural characteristics of patients in the two groups are summarized in Table 1.

In the TAVR group, the transfemoral approach was used in 39/40 patients (97.5%) while in one patient the left subclavian approach was used. The TAVR valves implanted were Edward Sapien 3 (35/40, 87.5%) and Medtronic Core valve (5/40, 12.5%). In the SAVR group, all surgeries were performed open sternotomy with cardiopulmonary bypass and implanted 82.5% Edwards Lifesciences (PERIMOUNT Magna™ and INSPIRIS™ RESILIA valves), and 17.5% Medtronic (Mosaic™, Avalus Bovine Aortic Surgical Valves), based on surgeon preference.

### Aortic global circumferential strain (GCS)

The change in GCS (Delta GCS, pre vs. post-procedure GCS) was significant in the TAVR (median 10.7 [IQR 4.5, 14.6] vs. 17.0 [IQR 6.1, 20.9], *p *= 0.009) but not in in the SAVR group (4.4 [IQR 3.3, 5.3] vs. 4.7 [IQR 3.9, 5.6], *p *= 0.3) (Table 2); the delta GCS was significantly higher in the TAVR group compared to the SAVR group: 2.8% [IQR 1.4, 6] vs. 0.15% [IQR − 0.6, 1.5], *p *< 0.001 (Table 4). The percentage delta GCS was also significantly greater in the TAVR versus SAVR group (28.8% [IQR 14.6, 64.6] vs. 4.4% [IQR − 10.6, 56], *p *= 0.006). (Table 3).

**Table 2:** Descending Aortic Biomechanics Before and After Interventions

	SAVR (n = 40)			TAVR (n = 40)		
	Pre	Post	*p*	Pre	Post	*p*
Global circumferential strain, GCS [%]	4.4 [3.3,5.3]	4.7 [3.9,5.6]	0.32	10.7 [4.5,14.6]	17.0 [6.1,20.9]	0.009
Pulse pressure corrected strain [%/mm Hg] (GCS/PP)	6.8 [5.5,9.2]	7.6 [6.1,10.8]	0.20	13.6 [8.6,23.5]	26.8 [10.6,32.0]	0.012
Time to peak strain, TTP [ms]	300 [270,334]	195 [167,250]	< 0.001	315 [285,400]	300 [280,385]	0.33
Δ Αrea/TTP [cm^2^/s]	1.3 [0.9,1.9]	2.5 [1.8,3.1]	< 0.001	2.2 [1.5,3.7]	2.6 [1.8,4.2]	0.22
Distensibility [10^–3^ mmHg^−1^]	1.5 [1.1,2.0]	1.4 [1.1,1.7]	0.52	3.6 [1.9,6.7]	4.9 [1.4,7.6]	0.76
Δ Αrea [cm^2^]	0.4 [0.3,0.6]	0.5 [0.4,0.6]	0.11	0.9 [0.4,1.2]	0.7 [0.6,1.4]	0.53
Fractional area change [%]	9.4 [6.8,11.9]	10.5 [9.3,13.5]	0.09	30.0 [9.1,40.8]	32.9 [103,57.1]	0.22
End systolic area [cm^2^]	5.3 [4.1,5.9]	4.8 [4.1,6.0]	0.81	4.6 [3.7,5.6]	4.4 [3.3,5.7]	0.90
End diastolic area [cm^2^]	4.8 [3.7,5.7]	4.3 [3.6,5.4]	0.65	3.3 [2.6,4.9]	3.3 [2.5,4.7]	0.76

*cm* centimeters, *GCS* Global circumferential strain [%], *ms* milliseconds, *PP* pulse pressure, *SAVR* surgical aortic valve replacement, *TAVR* transcatheter aortic valve replacement, *TTP*, time to peak

**Table 3:** Absolute (delta) and relative (percentage change) differences before and after interventions after adjustment for key variables

	Overall (n = 80)	SAVR (n = 40)	TAVR (n = 40)	*P*
Delta GCS	1.5 [− 0.1,3.4]	0.15 [− 0.6,1.5]	2.8 [1.4,6]	< 0.001
Delta GCS/PP	3.05 [− 0.01,7.7]	1.3 [− 1.5,3.2]	6.1 [1.6,12.3]	< 0.001
Delta GCS (Mean (SE)) *	–	0.2 (0.9)	4.2 (0.9)	0.011
Delta GCS/PP (Mean (SE)) *	–	0.5(2.1)	9.2 (2.1)	0.024
Percent change GCS	21.2 [− 2.3,58]	4.4 [− 10.6,56]	28.8 [14.6,64.6]	0.006
Percent change GCS (GCS/PP)	31.9 [0.14,78.8]	18.4 [− 27.4,46.4]	36.9 [11.2,83]	0.11
Percent change GCS ((Mean (SE)) *	–	− 1.8 (13.3)	62.0 (13.3)	0.010
Percent change GCS/PP (Mean (SE)) *	–	9.5 (18.4)	81.6 (18.4)	0.035
Delta EDA	− 0.1 [− 0.6,0.3]	− 0.04 [− 0.36,0.36]	− 0.25 [− 0.8,0.3]	0.37
Delta ESA	− 0.1 [− 0.5,0.5]	− 0.02 [− 0.36,0.6]	− 0.2 [− 0.7,0.26]	0.38
Delta FAC	1.6 [− 1.8,6.1]	1.3 [− 0.5,4.7]	1.9 [− 2.1,14.1]	0.45

All values were (median [interquartile range]) except for mean and SE

*ANCOVA Adjusted. *SE* standard error, *cm* centimeters, *EDA* End diastolic area, *ESA* End systolic area, *FAC* Fractional area change, *GCS* Global circumferential strain [%], *ms* Milliseconds, *PP* Pulse pressure, *SAVR* Surgical aortic valve replacement, *TAVR* Transcatheter aortic valve replacement, *TTP* Time to peak

The pulse-pressure corrected GCS was significantly higher post-procedure in the TAVR (13.6% [IQR 8.6, 23.5] vs. 26.8% [IQR 10.6, 32], *p *= 0.012) but not in the SAVR group (6.8% [IQR 5.5, 9.2] vs. 7.6% [IQR 6.1, 10.8], *p *= 0.2). The pulse-pressure corrected delta GCS was also significantly greater in the TAVR compared to the SAVR group (6.1% [IQR 1.6, 12.3] vs. 1.3% [IQR − 1.5, 3.2], *p *< 0.001). (Table 3).

**Fig. 3:**
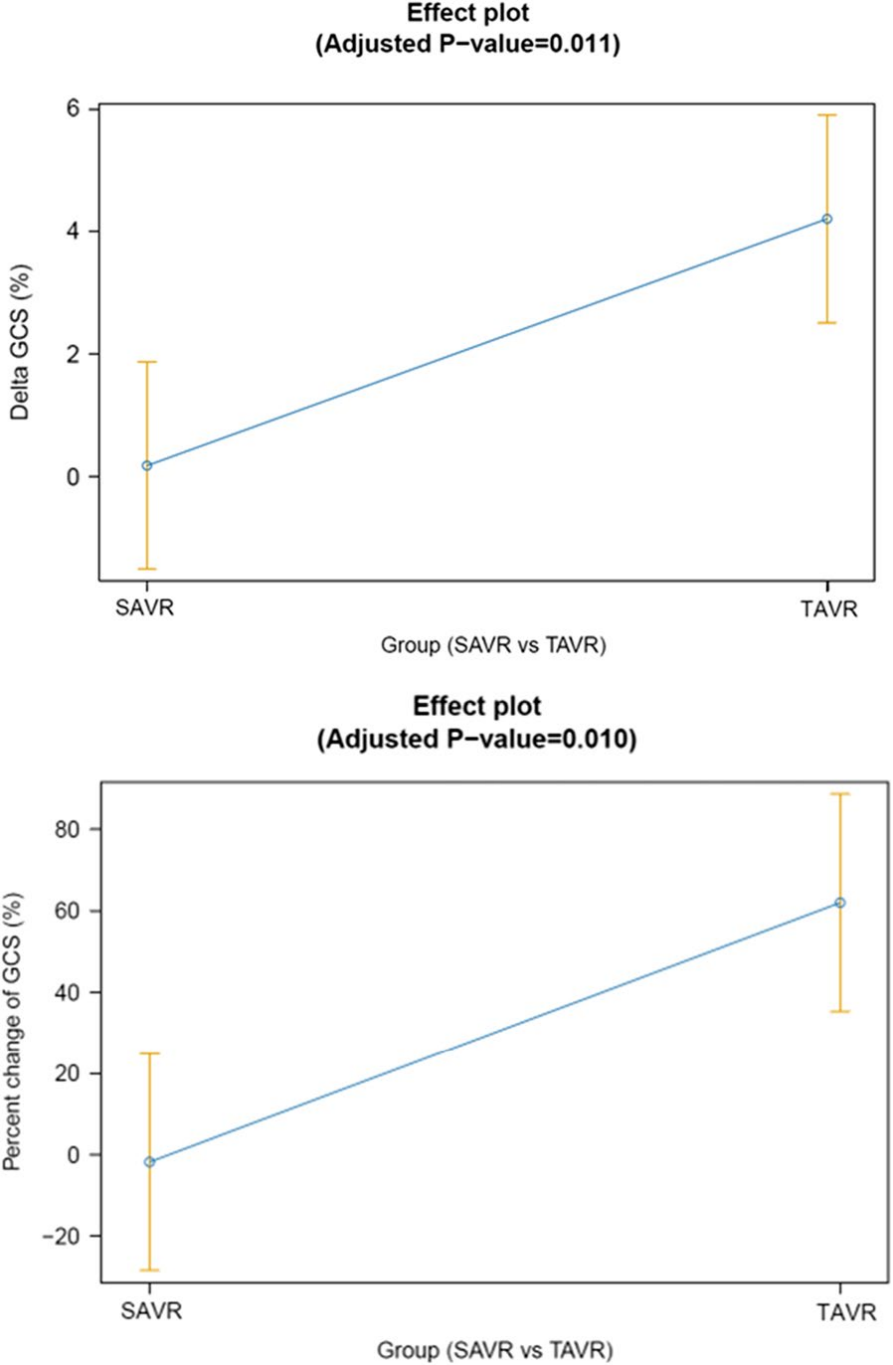
Effect plots showing **A** delta GCS (%) among TAVR versus SAVR after adjustment for key baseline and hemodynamic variables using ANCOVA and **B** percent change GCS (%) among AVR versus TAVR after adjustment for key baseline and hemodynamic variables using ANCOVA

In the fully adjusted ANCOVA model, the delta GCS and the percentage delta GCS were both significantly greater in the TAVR compared to the SAVR group (0.2% ± 0.8 vs. 4.2% ± 0.8, *p *< 0.001 and − 1.8% ± 13.3 vs. 62.0% ± 13.3, *p *< 0.010 respectively, Table 3, Fig. 3). The delta GCS/PP and percentage delta GCS/PP were also significantly greater in the TAVR versus SAVR groups (0.5% ± 2.1 vs. 9.2% ± 2.1), *p *< 0.024, and 9.5% ± 18.4) vs. 81.6% ± 18.4, *p *< 0.035).

In the sensitivity analysis excluding BAV patients, both delta GCS and percentage delta GCS remained significantly greater after TAVR versus SAVR before and after ANCOVA with adjustment for key variable (Additional file 1: Tables S1 and S2). In the sensitivity analysis adjusted by imaging modality, the results remained solid after accounting for imaging modality in the multivariable ANCOVA model (TEE vs. TTE) (Additional file 1: Table S3).

### Other measures

Post-procedure TTP and distensibility were significantly higher in the TAVR group (300 ms [IQR 280, 385] vs. 195 ms [IQR 166.8, 250], and 4.9 (10^−3^ mmHg) [IQR 1.4, 7.6] vs. 1.4 (10^−3^ mmHg) [IQR 1.1, 1.7], *p *< 0.001 for both—Table 4).

**Table 4:** Biomechanical variables before and after interventions

	Overall (n = 80)	SAVR (n = 40)	TAVR (n = 40)	*P*
*Pre-procedure strain variables*				
GCS (%)	5.2 [3.4,10.7]	4.4 [3.3,5.3]	10.7 [4.5,14.6]	< 0.001
Pulse pressure-corrected strain (GCS/PP)	9.1 [6.3,14.6]	6.8 [5.5,9.2]	13.6 [8.6,23.5]	< 0.001
TTP (ms)	300 [270,370.3]	300 [270,334]	315 [285,400]	0.14
ΔΑrea/TTP [cm^2^/s]	1.7 [1.04,2.5]	1.3 [0.9,1.9]	2.2 [1.5,3.7]	0.001
Distensibility (10^−3^ mmHg)	2 [1.3,4.2]	1.5 [1.1,2.04]	3.6 [1.9,6.7]	< 0.001
FAC (%)	10.7 [7.4,29.8]	9.35 [6.8,11.9]	30 [9.4,40.8]	< 0.001
ESA (cm^2^)	4.8 [3.8,5.7]	5.3 [4.07,5.9]	4.6 [3.7,5.6]	0.12
EDA (cm^2^)	4.1 [3.1,5.3]	4.8 [3.7,5.6]	3.3 [2.6,4.9]	0.002
*Post-procedure strain variables*				
GCS (%)	5.6 [4.1,16.23]	4.65 [3.9,5.6]	17 [6.1,20.9]	< 0.001
Pulse pressure-corrected strain (GCS/PP %)	10.8 [6.3,26.5]	7.6 [6.1,10.8]	26.8 [10.6,32]	< 0.001
TTP (ms)	263.5 [187.5,305]	195 [166.8,250]	300 [280,385]	< 0.001
Δ Αrea/TTP [cm^2^/s]	2.6 [1.8,3.6]	2.5 [1.8,3.1]	2.6 [1.8,4.2]	0.26
PP (mmHg)	61 [47.8,72.3]	60 [46.8,67.5]	63 [50.8,77]	0.08
Distensibility (10^−3^ mmHg)	1.6 [1.2,4.95]	1.4 [1.1,1.7]	4.9 [1.4,7.6]	< 0.001
FAC (%)	12.3 [9.5,35.2]	10.5 [9.3,13.5]	32.9 [10.3,57.1]	< 0.001
ESA (cm^2^)	4.6 [3.7,5.9]	4.8 [4.1,6.0]	4.3 [3.3,5.7]	0.14
EDA (cm^2^)	3.9 [2.9,5]	4.3 [3.6,5.4]	3.3 [2.5,4.7]	0.002

All values were (median [interquartile range])

*cm* Centimeters, *EDA* End diastolic area, *ESA* End systolic area, *FAC* Fractional area change, *GCS* Global circumferential strain [%], *ms* Milliseconds, *PP* Pulse pressure, *SAVR* Surgical aortic valve replacement, *TAVR* Transcatheter aortic valve replacement, *TTP* Time to peak

There were no significant differences between the pre and post-procedure delta GCS/PP, delta EDA, delta ESA, and delta FAC, delta area, or aortic ESA and conventional imaging variables between the TAVR and SAVR groups (Tables 3, 4).

Hemodynamics and measures of left ventricular function in the two groups at the different timepoints are presented in Table 5.

**Table 5:** Hemodynamic variables before and after interventions

	Overall (n = 80)	SAVR (n = 40)	TAVR (n = 40)	*P*
*Pre-procedure hemodynamics*				
HR (bpm) (median **[**IQR])	63.5 [56.7,72]	60 [55.75,70.25]	65.5 [59,73.2]	0.10
SBP (mmHg)	122 [110.5,138]	122 [110.2,134]	122 [110.5,145]	0.57
DBP (mmHg)	58 [52,65]	60 [52.7,65]	57 [51,64]	0.29
PP (mmHg)	63.5 [51.8,78]	61 [50,75]	69 [56, 79]	0.14
EDV (ml)	109.9 [87,132.7]	106.59 [80.8,119.4]	123.93 [91.85,163.9]	0.021
ESV (ml)	42.4 [31.02,57.02]	38.1 [27.9,49.8]	42.8 [35.08,69.16]	0.049
EF (%)	62.5 [55, 66.5]	60 [59.1,67.2]	62.5 [47.9,65.5]	0.46
SV (ml)	67.6 [53.02,80.2]	61.8 [52.3,72.68]	70 [58.27,84.2]	0.09
CO (L/min)	4.2 [3.4,5.06]	3.7 [3.16,4.7]	4.4 [3.7,5.6]	0.025
CI (L/min/m^2^)	2.3 [1.85,2.6]	2.03 [1.7,2.37]	2.5 [2.09,2.8]	< 0.001
*Post-procedure hemodynamics*				
HR (bpm) (median [IQR])	69.5 [63.7,80]	77 [69,84]	64.5 [60.7,71.5]	< 0.001
SBP (mmHg)	115.5 [103.7,131]	115.5 [102.7,129.5]	116 [104.75,132]	0.54
DBP (mmHg)	56 [51,62.5]	57.5 [51.75,66.5]	54.5 [49.5,60]	0.17
EDV (ml)	93.6 [74.9,130.4]	87.9 [65.32,101.5]	120.6 [90.67,141.05]	< 0.001
PP (mmHg)	61 [47.8,72.3]	60 [46.8,67.5]	63 [50.8,77]	0.08
ESV (ml)	25.8 [16.35,50.75]	21.2 [15.7,38.3]	34.3 [19.3,56.3]	0.059
EF (%)	67 [59,84]	69 [60,84.5]	66.5 [56.75,84]	0.69
SV (ml)	70.9 [54.76,83.7]	59.5 [46.16,73.8]	77.9 [66.6,96.5]	< 0.001
CO (L/min)	5.09 [4.07,6.1]	4.89 [3.9,5.89]	5.2 [4.38,6.1]	0.56
CI (L/min/m^2^)	2.7 [2.16,3.38]	2.67 [2.09,3.1]	2.8 [2.3,3.49]	0.14

All values were (median [interquartile range])

*bpm* Beats per minute, *CI* Cardiac index, *cm* Centimeters, *CO* Cardiac output, *DBP* Diastolic blood pressure, *EDV* End diastolic volume, *EF* Ejection fraction, *ESV* End systolic volume, *HR* Heart rate, *IQR* Inter-quartile range, *L/min* Liters/minute, m Meters, *ml* Milliliter, *mmHg* Millimeters mercury, *ms* Milliseconds, *SAVR* Surgical aortic valve replacement, *SBP* Systolic blood pressure, *TAVR* Transcatheter aortic valve replacement

## Discussion

This study found that the absolute and relative change in circumferential strain in the descending aorta after aortic valve interventions was significantly greater after TAVR than after SAVR. This was confirmed after adjustment for key variables including baseline GCS, age, sex, blood pressure, pre and post-procedure cardiac index and aortic valve gradient. Our hypothesis-generating results suggest that TAVR implantation is associated with increased energy propagation to the descending aorta and with a potentially increased risk of post-procedural adverse aortic events compared to SAVR. This hypothesis is clinically important given the ongoing trend to expand TAVR interventions to a younger population and to patients with BAV who are more susceptible to aortic events.

A study using the National Inpatient Sample found that the percentage of patients ≤ 65 years old undergoing TAVR increased from 2012 to 2015, from 1.2 to 3.5% in the ≤ 55-year group, and from 2.5 to 7.3% in the 55–65 year age group [13]. In another study based on the Transcatheter Valve Therapy Registry of over 160,000 patients, the proportion of patients at low surgical risk who underwent TAVR increased from 9.6% in 2015 to 43.8% in 2020 and the proportion of patients with BAV undergoing TAVR increased from 2.8% in 2015 to 6.8% in 2020 [14].

While previous studies have shown that the relative survival of patients with bicuspid AS who underwent SAVR is excellent and comparable to a matched general population, [15] no similar data exist for patients with BAV who undergo TAVR [15]. This study evaluating differential impact of TAVR versus SAVR on the aorta is important considering the known higher risk of aortic dissection and acute aortic events in BAV patients [16].

These findings also have implications for patients with concomitant ascending aortic aneurysms undergoing TAVR. One group reported that over 25% of their patients undergoing TAVR had ascending aortic aneurysms of 4 to 5 cm [17]. At a mean follow-up of 14 months, the authors found comparable intraprocedural safety between the aneurysm and non-aneurysm groups and found that ascending aortic diameters remained stable in their relatively old, non-BAV population. However, it is unclear if this would be true in younger patients or in a BAV population at longer follow-up. It should be considered that our data were recorded during sedation and anesthesia, and it is reasonable to believe that the difference in energy propagation between groups is magnified during exercise (an issue of particular relevance in the young and active patient population).

Circumferential strain has been used to study aortic physiology in cardiac surgical populations and allows for accurate quantification of arterial biomechanics [6–9]. Circumferential strain is the change or displacement of the circumference from its baseline value and is quantified by the percentage change in circumference between the aorta at baseline and during systolic deformation. Circumferential strain of the aorta from two-dimensional TEE or TTE echo images can be calculated over the circumference of the aortic wall in the short axis view over the cardiac cycle as a measure of energy propagation [10].

Previous studies have used GCS to investigate the acute impact of ascending aortic graft replacement on descending aorta biomechanics, and the effect on the distal aorta of SAVR for aortic stenosis versus aortic insufficiency [6]. Other studies have shown that flow patterns in the ascending aorta after SAVR may be different using different aortic valve prostheses [18]. A study by Bisell et al. evaluated flow patterns and wall stress on the proximal aorta after mechanical versus bioprosthetic SAVR and found decreased wall stress with mechanical valves and suggested this as potential mechanism for future aneurysm formation [19].

There are several potential reasons why TAVR valves may result in greater energy propagation to the distal aorta compared to SAVR. One is that TAVR devices, either self-expanding or balloon-expandable, differ from surgical valves by containing a valve within a metal frame, which serves to anchor the device in the annulus and proximal ascending aorta without sutures. These caged prostheses differ from the native aorta in geometry and compliance, and likely interfere with the pressure-regulating effect of the ascending aorta (Windkessel effect) alter pulse wave propagation and cause high velocity flow to distal segments of the aorta, providing potential for adverse remodeling and dissection [20, 21]. Another possible reason is that TAVR valves have a lower profile and increased effective orifice areas compared to surgical valves, allowing higher stroke volume [22] and distal energy propagation.

The possibility of a different effect of TAVR versus SAVR on post-procedural aortic biomechanics and potentially on the risk of aneurysm development among patients with AS is a clinically important question in an era in which TAVR indications expand to patients with bicuspid aortic valve and to young patients with long life expectancy. The increase in distal energy propagation after TAVR versus SAVR may accelerate aneurysm growth, especially in high-risk populations and may require dedicated follow-up imaging surveillance after the procedure [23].

This study has several limitations. First, this is a hypothesis-generating study, and no formal sample size calculation was performed. The TAVR group used mostly TTE to evaluate the differences in GCS in the descending aorta while the SAVR group used TEE. While both have been validated for imaging of the descending aorta, we addressed this potential confounder by evaluating the relative percentage change in strain and using ANCOVA, a statistical approach that accounts for different baseline aortic strain values. The two patient groups were different in a number of variables at baseline and intraprocedural timepoints. However, we adjusted for all key baseline clinical and hemodynamic variables and found that delta GCS and the percentage change in delta GCS remained significantly greater in the TAVR group. We also performed a sensitivity analysis accounting for imaging modality in the multivariable model (TEE vs. TTE) which supported our primary results. Also, the absolute differences in GCS between groups were large, and unlikely explained only by unaccounted confounders. Finally, our findings are limited to the time immediately post-procedure and further studies are needed to longitudinally explore the changes in the distal aorta after TAVR versus SAVR.

## Conclusions

This is the first study to evaluate distal aortic biomechanics after TAVR vs. SAVR. We found that the increase in post-procedure energy propagation to the distal aorta was significantly greater after TAVR even after adjustment for important confounders.

These findings suggest that TAVR has differential downstream effects on aortic deformation and flow compared to SAVR and this may have potentially important implications for the risk of post-procedural aortic events, especially in high-risk categories of patients. Due to the growing adoption of TAVR in young patients and in patients with BAV, further studies on this topic are urgently needed.

### Supplementary Information

**Additional file 1: Table S1.** Descending Aortic Biomechanics Before and After Interventions excluding BAV patients. **Table S2.** Absolute (Delta) and Relative (Percentage Change) Differences Before and After Interventions after adjustment for key variables excluding BAV patients. **Table S3. **Absolute (Delta) and Relative (Percentage Change) Differences Before and After Interventions after addition of imaging modality to the model. **Table S4.** Abbreviations.

## Data Availability

The data used to support the findings of this study are available from the corresponding author upon request.

## References

[1] Makkar RR, Thourani VH, Mack MJ, Kodali SK, Kapadia S, Webb JG (2020). Five-year outcomes of transcatheter or surgical aortic-valve replacement. N Engl J Med.

[2] Popma JJ, Deeb GM, Yakubov SJ, Mumtaz M, Gada H, O’Hair D (2019). Transcatheter aortic-valve replacement with a self-expanding valve in low-risk patients. N Engl J Med.

[3] Reardon MJ, Van Mieghem NM, Popma JJ, Kleiman NS, Søndergaard L, Mumtaz M (2017). Surgical or transcatheter aortic-valve replacement in intermediate-risk patients. N Engl J Med.

[4] Smith CR, Leon MB, Mack MJ, Miller DC, Moses JW, Svensson LG (2011). Transcatheter versus surgical aortic-valve replacement in high-risk patients. N Engl J Med.

[5] Mack MJ, Leon MB, Thourani VH, Makkar R, Kodali SK, Russo M (2019). Transcatheter aortic-valve replacement with a balloon-expandable valve in low-risk patients. N Engl J Med.

[6] Rong LQ, Palumbo MC, Rahouma M, Devereux RB, Kim J, Pryor KO (2021). Differential effects of aortic valve replacement on aortic circumferential strain in aortic stenosis and aortic insufficiency. J Cardiothorac Vasc Anesth.

[7] Rong LQ, Palumbo MC, Rahouma M, Lopes AJ, Devereux RB, Kim J (2020). Descending aortic strain quantification by intra-operative transesophageal echocardiography: multimodality validation via cardiovascular magnetic resonance. Echocardiography.

[8] Palumbo MC, Rong LQ, Kim J, Navid P, Sultana R, Butcher J (2020). Prosthetic aortic graft replacement of the ascending thoracic aorta alters biomechanics of the native descending aorta as assessed by transthoracic echocardiography. PLoS ONE.

[9] Rong LQ, Palumbo MC, Rahouma MM, Meineri M, Arguelles GR, Kim J (2019). Immediate of impact prosthetic graft replacement of the ascending aorta on circumferential strain in the descending aorta. J Vasc Surg.

[10] Rong LQ, Kim J, Gregory AJ (2020). Speckle tracking echocardiography: imaging insights into the aorta. Curr Opin Cardiol.

[11] Alreshidan M, Shahmansouri N, Chung J, Lash V, Emmott A, Leask RL, Lachapelle K (2017). Obtaining the biomechanical behavior of ascending aortic aneurysm via the use of novel speckle tracking echocardiography. J Thorac Cardiovasc Surg.

[12] Voges I, Jerosch-Herold M, Hedderich J, Pardun E, Hart C, Gabbert DD (2012). Normal values of aortic dimensions, distensibility, and pulse wave velocity in children and young adults: a cross-sectional study. J Cardiovasc Magn Reson.

[13] Sedrakyan A, Dhruva SS, Sun T, Mao J, Gaudino MFL, Redberg RF (2018). Trends in use of transcatheter aortic valve replacement by age. JAMA.

[14] Makkar RR, Yoon S-H, Chakravarty T, Kapadia SR, Krishnaswamy A, Shah PB (2021). Association between transcatheter aortic valve replacement for bicuspid vs tricuspid aortic stenosis and mortality or stroke among patients at low surgical risk. JAMA.

[15] Glaser N, Jackson V, Eriksson P, Sartipy U, Franco-Cereceda A (2021). Relative survival after aortic valve surgery in patients with bicuspid aortic valves. Heart.

[16] Michelena HI, Khanna AD, Mahoney D, Margaryan E, Topilsky Y, Suri RM (2011). Incidence of aortic complications in patients with bicuspid aortic valves. JAMA.

[17] Rylski B, Szeto WY, Bavaria JE, Walsh E, Anwaruddin S, Desai ND (2014). Transcatheter aortic valve implantation in patients with ascending aortic dilatation: safety of the procedure and mid-term follow-up. Eur J Cardiothorac Surg.

[18] von Knobelsdorff-Brenkenhoff F, Trauzeddel RF, Barker AJ, Gruettner H, Markl M, Schulz-Menger J (2014). Blood flow characteristics in the ascending aorta after aortic valve replacement–a pilot study using 4D-flow MRI. Int J Cardiol.

[19] Bissell MM, Loudon M, Hess AT, Stoll V, Orchard E, Neubauer S, Myerson SG (2018). Differential flow improvements after valve replacements in bicuspid aortic valve disease: a cardiovascular magnetic resonance assessment. J Cardiovasc Magn Reson.

[20] Galea N, Piatti F, Sturla F, Weinsaft JW, Lau C, Chirichilli I (2018). Novel insights by 4D Flow imaging on aortic flow physiology after valve-sparing root replacement with or without neosinuses†. Interact Cardiovasc Thorac Surg.

[21] Cave DGW, Panayiotou H, Bissell MM (2021). Hemodynamic profiles before and after surgery in bicuspid aortic valve disease—a systematic review of the literature. Front Cardiovasc Med.

[22] Søndergaard L, Ihlemann N, Capodanno D, Jørgensen TH, Nissen H, Kjeldsen BJ (2019). Durability of transcatheter and surgical bioprosthetic aortic valves in patients at lower surgical risk. J Am Coll Cardiol.

[23] Spadaccio C, Nappi F, Al-Attar N, Sutherland FW, Acar C, Nenna A (2016). Old myths, new concerns: the long-term effects of ascending aorta replacement with dacron grafts. Not all that glitters is gold. J Cardiovasc Transl Res.

