# Supplementary material for: Distal aortic biomechanics after transcatheter versus surgical aortic valve replacement: a hypothesis generating study
Source: J Cardiothorac Surg. 2023 Nov 30;18:349. doi: 10.1186/s13019-023-02467-z (PMC10690972; doi:10.1186/s13019-023-02467-z)
Supplement: Supplementary file 1 — Additional file 1: Table S1. Descending Aortic Biomechanics Before and After Interventions excluding BAV patients. Table S2. Absolute (Delta) and Relative (Percentage Change) Differences Before and After Interventions after adjustment for key variables excluding BAV patients. Table S3. Absolute (Delta) and Relative (Percentage Change) Differences Before and After Interventions after addition of imaging modality to the model. Table S4. Abbreviations. [file 13019_2023_2467_MOESM1_ESM.docx]

**Supplemental Material:**

**Supplemental Table 1.** Descending Aortic Biomechanics Before and After Interventions excluding BAV patients

**Supplemental Table 2**: Absolute (Delta) and Relative (Percentage Change) Differences Before and After Interventions after adjustment for key variables excluding BAV patients.

**Supplemental Table 3:** Absolute (Delta) and Relative (Percentage Change) Differences Before and After Interventions after addition of imaging modality to the model

**Supplemental Table 4**. Abbreviations

**Supplemental Table 1.** Descending Aortic Biomechanics Before and After Interventions excluding BAV patients

|  | SAVR (n=27) |  |  | TAVR (n=37) |  |  |
| --- | --- | --- | --- | --- | --- | --- |
|  | Pre | Post | p | Pre | Post | p |
| Global circumferential strain, GCS [%] | 4.40 [3.80, 5.30] | 3.80 [3.05, 5.00] | 0.243 | 10.60 [4.30, 14.60] | 15.50 [5.60, 20.90] | 0.012 |
| Pulse Pressure Corrected Strain [%/mm Hg](GCS/PP) | 6.96 [6.14, 9.55] | 6.33 [5.28, 8.55] | 0.186 | 13.61 [8.67, 21.08] | 26.07 [9.03, 30.59] | 0.018 |

**Table Legend:** GCS, Global circumferential strain [%]; PP, pulse pressure; SAVR, surgical aortic valve replacement; TAVR, transcatheter aortic valve replacement. All values were (median [interquartile range]) except for mean and SE.

**Supplemental Table 2.** Absolute (Delta) and Relative (Percentage Change) Differences Before and After Interventions after adjustment for key variables excluding BAV patients.

|  | Overall  (n=64) | SAVR  (n=27) | TAVR  (n=37) | P |
| --- | --- | --- | --- | --- |
| Delta GCS | 1.60 [0.10, 3.75] | 0.30 [-0.85, 1.50] | 2.80 [1.30, 5.80] | <0.001 |
| Delta GCS/PP | 3.06 [-0.01, 7.33] | 1.53 [-0.74, 3.12] | 5.90 [0.23, 10.41] | 0.001 |
| Delta GCS (Mean (SE)) * | ---- | -0.04 (1.20) | 4.39 (0.92) | 0.026 |
| Delta GCS/PP (Mean (SE)) * | ---- | -0.60 (2.91) | 9.28 (2.22) | 0.039 |
| Percent Change GCS | 24.56 [3.04, 61.08] | 10.34 [-15.30, 56.35] | 29.27 [11.23, 65.38] | 0.038 |
| Percent Change GCS/PP | 31.54 [0.14, 75.90] | 27.86 [-11.64, 57.17] | 33.79 [3.47, 82.60] | 0.168 |
| Percent Change GCS ((Mean (SE)) * | ----- | -4.46 (18.53) | 62.20 (14.19) | 0.029 |
| Percent Change GCS/PP (Mean (SE)) * | ----- | 0.47 (25.30) | 80.09 (19.37) | 0.055 |

**Table Legend:** *ANCOVA adjusted, GCS, Global circumferential strain [%]; PP, pulse pressure; SE, standard error; SAVR, surgical aortic valve replacement; TAVR, transcatheter aortic valve replacement. All values were (median [interquartile range]) except for mean and SE.

**Supplemental Table 3**. Absolute (Delta) and Relative (Percentage Change) Differences Before and After Interventions after addition of imaging modality to the model

| All cases (without TEE/TTE in adjustment) | SAVR  (n=40) | TAVR  (n=40) | P |
| --- | --- | --- | --- |
| - Delta GCS (Mean (SE)) | 0.18 (0.9) | 4.2 (0.9) | 0.011 |
| - Delta GCS/PP (Mean (SE)) | 0.5 (2.1) | 9.2 (2.1) | 0.024 |
| - Percent Change GCS ((Mean (SE)) | -1.8 (13.3) | 62.0 (13.3) | 0.010 |
| - Percent Change GCS/PP (Mean (SE)) | 9.5 (18.4) | 81.6 (18.4) | 0.035 |
| All cases (Including TEE/TTE in adjustment) |  |  |  |
| - Delta GCS (Mean (SE)) | 1.05 (0.61) | 3.33 (0.61) | 0.044 |
| - Delta GCS/PP (Mean (SE)) | 2.4 (1.7) | 7.35 (1.67) | 0.11 |
| - Percent Change GCS (Mean (SE)) | 0.09 (0.1) | 0.51 (0.11) | 0.044 |
| - Percent Change GCS/PP (Mean (SE)) | 24.04 (15.9) | 67.06 (15.9) | 0.14 |

**Table Legend:** GCS, Global circumferential strain [%]; PP, pulse pressure; SAVR, surgical aortic valve replacement; SE, standard error; TAVR, transcatheter aortic valve replacement; TEE: Transesophageal echocardiogram; TTE: transthoracic echocardiogram.

**Supplemental Table 4**. Abbreviations

| ANCOVA | Analysis of covariance |
| --- | --- |
| BAV | Bicuspid aortic valve |
| CI | Cardiac Index |
| cm | Centimeter |
| CO | Cardiac output |
| EDA | End diastolic area |
| ESA | End systolic area |
| FAC | Fractional area change |
| GCS | Global circumferential strain |
| HR | Heart rate |
| IQR | Interquartile ranges |
| ms | Milliseconds |
| PP | Pulse pressure |
| SAVR | Surgical aortic valve replacement |
| SD | Standard deviation |
| TAVR | Transcatheter aortic replacement |
| TEE | Transesophageal echocardiography |
| TTE | Transthoracic echocardiography |
| TTP | Time to peak strain |
